# In vivo behaviour of a biodegradable poly(trimethylene carbonate) barrier membrane: a histological study in rats

**DOI:** 10.1007/s10856-012-4663-x

**Published:** 2012-05-09

**Authors:** A. C. Van Leeuwen, T. G. Van Kooten, D. W. Grijpma, R. R. M. Bos

**Affiliations:** 1 Department of Oral and Maxillofacial Surgery, University Medical Center Groningen, PO Box 30001, 9700 RB Groningen, The Netherlands; 2 Department of Biomedical Engineering, University Medical Center Groningen and University of Groningen, PO Box 196, 9700 AD Groningen, The Netherlands; 3 MIRA Institute for Biomedical Engineering and Technical Medicine, and Department of Biomaterials Science and Technology, Faculty of Science and Technology, University of Twente, PO Box 217, 7500 AE Enschede, The Netherlands

## Abstract

The aim of the present study was to evaluate the response of surrounding tissues to newly developed poly(trimethylene carbonate) (PTMC) membranes. Furthermore, the tissue formation beneath and the space maintaining properties of the PTMC membrane were evaluated. Results were compared with a collagen membrane (Geistlich BioGide), which served as control. Single-sided standardized 5.0 mm circular bicortical defects were created in the mandibular angle of rats. Defects were covered with either the PTMC membrane or a collagen membrane. After 2, 4 and 12 weeks rats were sacrificed and histology was performed. The PTMC membranes induced a mild tissue reaction corresponding to a normal foreign body reaction. The PTMC membranes showed minimal cellular capsule formation and showed signs of a surface erosion process. Bone tissue formed beneath the PTMC membranes comparable to that beneath the collagen membranes. The space maintaining properties of the PTMC membranes were superior to those of the collagen membrane. Newly developed PTMC membranes can be used with success as barrier membranes in critical size rat mandibular defects.

## Introduction

Guided bone regeneration (GBR) is a widely used modality for restoring bone deficiencies. In GBR the use of barrier membranes leads to predictable bone formation, by preventing in-growth of fibroblasts and provision of space for osteogenesis within a blood clot formed in the defect [1]. A variety of biocompatible membranes have been used to achieve this desired barrier effect. The optimal barrier membrane should exert biocompatible, synthetic, degradable and space maintaining properties [2, 3]. Currently, non-resorbable membranes have better space maintaining properties compared to resorbable membranes. However, a major disadvantage of non-resorbable membranes is the need for their removal in a second operation.

The majority of clinically used resorbable membranes are based on collagen. As collagen is an animal derived product, these membranes carry the risk of disease transmission from animal to human [3–5]. Other available resorbable barrier membranes are synthetic polymeric membranes based on lactide and glycolide. However, due to an extensive foreign body reaction, adverse effects like postoperative swelling have been reported when using these materials [6–13]. It is known that these materials can produce significant amounts of acidic compounds during degradation in the body, and since bone dissolves in acidic environments, it can be expected that these polymers will not be the most suited materials for use in GBR [7, 12, 14, 15].

Recently, we have developed a flexible synthetic biodegradable barrier membrane prepared from poly(trimethylene carbonate) (PTMC) for GBR [16]. PTMC is a flexible, rubber-like, amorphous and biodegradable polymer. The monomer from which it is prepared, trimethylene carbonate (TMC), has been used to prepare copolymers for use in barrier membranes in the medical field before [17–20]. By gamma irradiation under vacuum, form-stable elastomeric networks can be formed [21]. In vitro and in vivo research has shown that this polymer is both biocompatible and can be degraded by surface erosion without the formation of acidic degradation products [22, 23].

In the aforementioned study [16] the PTMC membrane, prepared from TMC only, was evaluated and compared with a collagen (Geistlich BioGide) and an expanded-polytetrafluoroethylene (e-PTFE, GoreTex) membrane and a non-treated control site for its suitability as a barrier membrane for use in GBR over bony defects in rats. Both quantitative micro-radiographical and quantitative micro-computed tomographical analysis showed excellent amounts of bone formed underneath the PTMC membrane [16]. Evaluation after 12 weeks showed that comparable amounts of bone had formed underneath the PTMC and reference membranes. The mean percentages of regenerated bone were 74, 71, 83 and 33 %, for respectively the collagen, PTMC e-PTFE and the non-treated control group [16].

The purpose of this current study was to evaluate histologically the response of the surrounding tissue to the PTMC membrane, the tissue formation beneath the PTMC membrane, and the space maintaining properties of the PTMC membrane. Furthermore, the degradation of the membrane was assessed. The collagen membrane served as a reference.

## Materials and methods

### Membrane synthesis

Polymerization grade 1,3-trimethylene carbonate (TMC) was obtained from Boehringer Ingelheim, Germany. Stannous octoate (SnOct_2_ from Sigma, USA) was used as received. The used solvents were of analytical grade and purchased from Biosolve, The Netherlands. PTMC was synthesized by ring opening polymerization of TMC monomer under vacuum at 130 °C for a period of three days using stannous octoate as a catalyst. The polymer was purified by dissolution in chloroform and precipitation into a fivefold excess of ethanol 100 %. The precipitated PTMC was dried under vacuum at room temperature. Analysis of the synthesized polymer by proton nuclear magnetic resonance (^1^H NMR), gel permeation chromatography (GPC) and differential scanning calorimetry (DSC) according to standardized procedures [21] indicated that high molecular weight polymer had been synthesized. GPC measurements showed that M_*w*_ = 443000 and M_*n*_ = 332000 g/mol, while NMR indicated that the monomer conversion was more than 98 %. The glass transition temperature of this amorphous polymer was approximately −17 °C, as thermal analysis showed.

The purified polymer was then compression moulded at 140 °C and a pressure of 3.0 MPa (31 kg/cm^2^) using a Carver model 3851-0 laboratory press (Carver Inc, USA) into films with a thickness of 0.3 mm and a diameter of 8 mm using stainless steel moulds. The PTMC membranes were then sealed under vacuum and exposed to 25 kGy gamma irradiation from a ^60^Co source (Isotron BV, The Netherlands). This sterilization method leads to simultaneous crosslinking of the polymer [21].

### Study design

The gamma irradiated PTMC membranes were evaluated by a subperiosteal implantation in rats. All procedures performed on the animals were done according to international standards on animal welfare and complied with the Animal Research Committee of the University Medical Centre Groningen. Twenty-four Sprague–Dawley rats from an earlier study [16] were included in here. The surgical procedure consisted of drilling one bicortical critical size (5.0 mm diameter) mandibular defect in the left mandibular angle with a trephine. The created defects were covered with a barrier membrane on the buccal and lingual side using either the PTMC membrane, or a collagen (Geistlich BioGide, Geistlich, Switzerland) membrane which served as reference. (The procedures are described in the section “Implantation procedure”*.*) At three different time periods (2, 4 and 12 weeks) the rats were sacrificed. Per time period eight rats were evaluated; four rats treated with the collagen membrane and four rats treated with the PTMC membrane. The rats were histologically evaluated for the response of the surrounding tissue to the membranes, and the tissue proliferation at the defect site, covered by the membranes. Furthermore, space maintaining properties and degradation of the PTMC membranes were assessed. 

(In a separate study, of which the results will be reported in another communication, the bone sample that was obtained with the trephine was transplanted to the contralateral mandibular angle to evaluate the suitability of the membranes on modelling and incorporation of autologous bone grafts.)

### Implantation procedure

The animals were anaesthetized with nitrous–oxygen–isoflurane. After the mandibular area was shaved and disinfected, a peri angular incision was made and a standardised circular 5.0 mm critical size bicortical defect was drilled with a trephine in the left mandibular angle [24, 25]. In one group the PTMC membrane was used to cover the defects. In the other group the defects were covered with a collagen membrane (Geistlich BioGide, Geistlich, Switzerland). The wound was closed in layers using resorbable sutures (Vicryl Rapide 4-0, Ethicon, USA). A single dose of Temgesic (0.05 mg/kg) was administered perioperative for postoperative pain relief, and the diet was composed of standard laboratory food.

At three different time intervals (2, 4 and 12 weeks), the rats were anaesthetised by nitrous–oxygen–isoflurane inhalation and sacrificed by an intracardially injected overdose of pentobarbital. The mandibles were explanted and fixed in 4 % phosphate buffered formaline solution.

### Light microscopy: histological scoring analysis

After explantation, the specimens were decalcified and dehydrated in a series of ethanol. The specimens were embedded in glycidyl methacrylate. The tissue blocks were cut perpendicularly to the defects with a microtome. The resulting histological sections were 2 μm thick. From all samples two sections were stained, one series with toluidine blue and one with toluidine blue/basic fuchsin. The histological sections were digitalized with a ScanScope GL (Aperio Technology Inc., Vista, CA). Digital images were then stored for further analysis. Histological sections were qualitatively and semiquantitatively analyzed using a modified histological scoring analysis (Table 1). Two histological sections were evaluated for each animal. The investigators were blinded for time interval and type of material used during evaluation of the explanted samples. During analysis each section was evaluated with respect to the foreign body response (soft tissue response to PTMC membrane), (the kind of) tissue proliferation at the defect site and finally for the space maintaining properties of the membrane. Each section received a single score. The scores for each time interval were then averaged; mean and standard deviation were reported.

**Table 1:** Semiquantitative histological grading scale

Soft tissue response to membrane	
Fibrous, mature, not dense, resembling connective or at tissue in the noninjured regions	4
Shows blood vessels and young fibroblasts, few macrophages and giant cells are present	3
Shows macrophages and other inflammatory cells in abundance, but connective tissue components between	2
Dense and exclusively of inflammatory type	1
Cannot be evaluated because of infection or other factors not necessarily related to the material	0
Tissue proliferation in defect	
Mature bone and differentiation of bone marrow	4
Bone or osteoid formation	3
Fibrous connective tissue: collagen fibers at defect site	2
Fibrous connective tissue: cellular and vascular components	1
Cannot be evaluated because of infection or other factors not necessarily related to the material	0
Space maintaining properties of membrane	
No contact between membranes at defect site, bone formation in between	4
No contact between membranes at defect site, connective tissue in between	3
Contact between membranes at defect site, bone formation present	2
Contact between membranes at defect site, connective tissue in between	1
Cannot be evaluated because of degradation or absence of the material	0

### Statistical analysis

The data sets were statistically evaluated with a Fisher’s Exact Test using SPSS 17 (Statistical Package for the Social Sciences, SPSS Inc., USA). The null hypothesis (the means of each set are equal) was evaluated with 95 % confidence level (α = 0.05).

## Results

### Clinical observations

None of the rats died or suffered from postoperative complications. The animals started eating immediately after the surgical procedure and did not loose weight.

### Descriptive microscopic observations

**Fig. 1:**
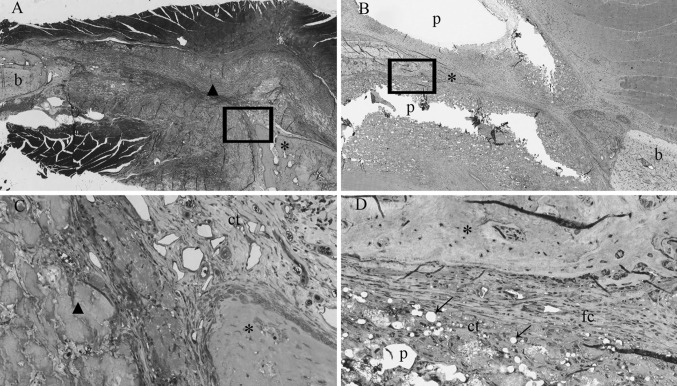
Light micrographs of rat mandibular defects covered with either a collagen (**a**, **c**) or a PTMC (**b**, **d**) membrane after 2 weeks of implantation. The collagen and PTMC membrane can be readily distinguished. Degradation of the PTMC membrane can be observed. Toluidin blue with basic fuchsin as counterstain was used for the staining of the histological sections. (*p*) polymer, (*ct*) connective tissue, (*b*) bone, (*fc*) fibrous capsule, (*asterisk*) osteoid, (*filled triangle*) collagen membrane, *arrows* indicate phagocytosed intracellular fragments. Figures ‘**c** and **d**’ magnifications (×20) of the marked regions from respectively figures ‘**a** and **b**’, which are ×2 magnifications

**Fig. 2:**
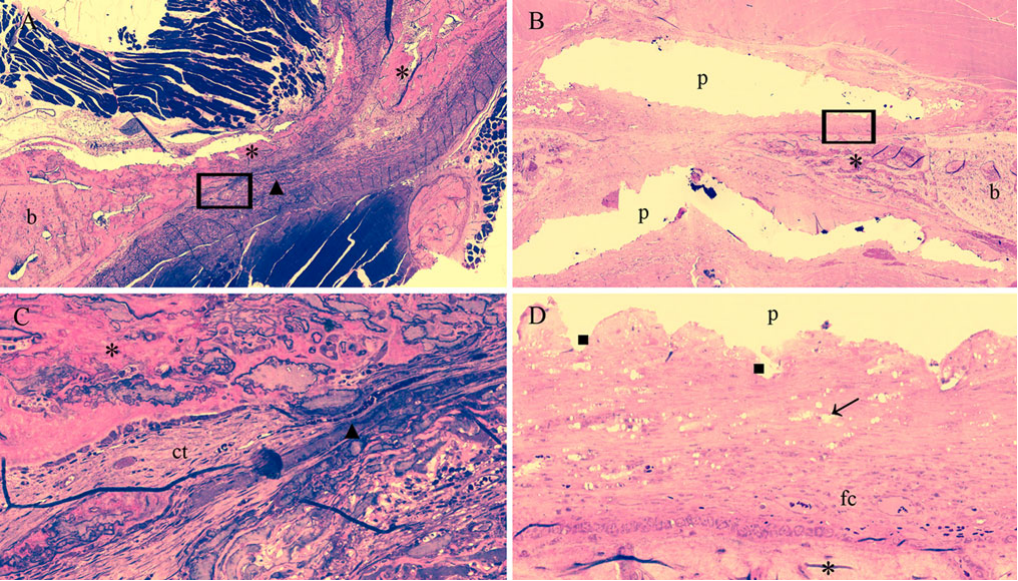
Light micrographs of rat mandibular defects covered with either a collagen (**a**, **c**) or a PTMC (**b**, **d**) membrane after 4 weeks of implantation. The collagen membrane can be distinguished from the rats connective tissue. Bone forms in and around the collagen membrane, by contrats bone formation underneath the PTMC membrane originates from the defect borders. Toluidin blue with basic fuchsin as counterstain was used for the staining of the histological sections. (*p*) polymer, (*ct*) connective tissue, (*b*) bone, (*fc*) fibrous capsule, (*asterisk*) osteoid, (*filled triangle*) collagen membrane, *arrows* indicate phagocytosed intracellular fragments. (*filled square*) Indicates surface erosion degradation by macrophage and giant cell activity. Figures ‘**c** and **d**’ magnifications (×20) of the marked regions from respectively figures ‘**a** and **b**’, which are ×2 magnifications

A gross light microscopical inspection of all histological sections was performed. Defects and the PTMC and collagen barrier membranes could easily be identified after 2–4 weeks (Figs. 1, 2). The PTMC membrane appeared white in the sections, the collagen barrier membrane appeared similar to (collagen) connective tissue, although it could be differentiated from host (collagen) connective tissue.

**Fig. 3:**
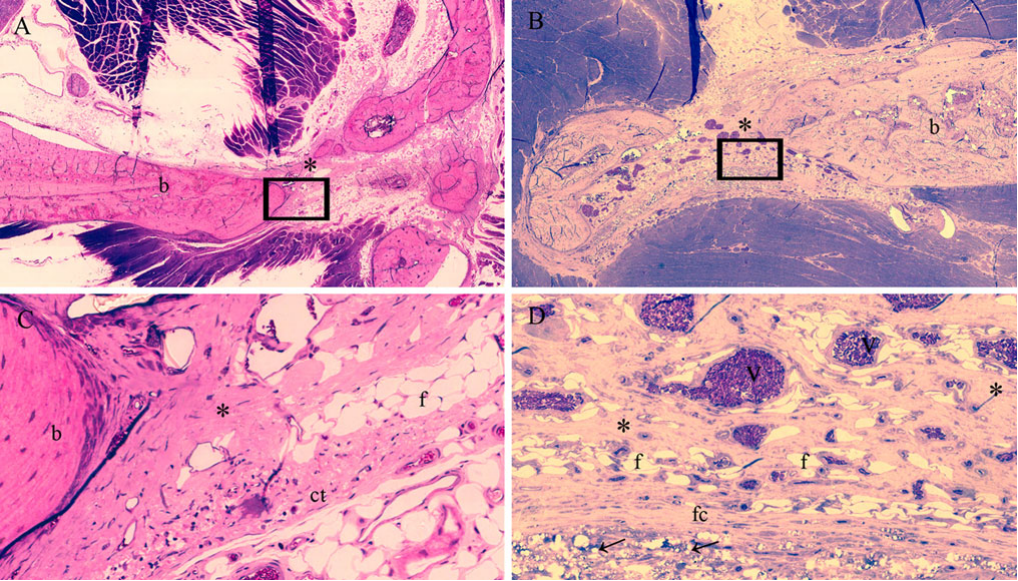
Light micrographs of rat mandibular defects covered with either a collagen (**a**, **c**) or a PTMC (**b**, **d**) membrane after 12 weeks of implantation. Bone bridges the mandibular defects in both membrane groups. The collagen membrane is not present anymore and has resorbed. The PTMC membrane eroded completely, only a few phagocytosed polymer particles are in situ. Toluidin blue with basic fuchsin as counterstain was used for the staining of the histological sections. (*p*) polymer, (*ct*) connective tissue, (*b*) bone, (*fc*) fibrous capsule, (*asterisk*) osteoid, (*v*) marks blood vessel, (*f*) fat cell, *arrows* indicate phagocytosed intracellular fragments. Figures ‘**c** and **d**’ are magnifications (×20) of the marked regions from respectively figures ‘**a** and **b**’, which are ×2 magnifications

At 12 weeks it was more difficult to identify the defects, whereas in some animals the defect had closed. The collagen membrane could not be identified anymore at 12 weeks because, besides extensive signs of degradation of the membrane, the differentiation between membrane and host collagen was virtually impossible. The PTMC membrane showed extensive signs of degradation. Only some remnants of the PTMC membrane could still be identified after 12 weeks (Fig. 3).

After 2 weeks both the collagen membrane and the PTMC membrane was surrounded by a thin fibrous capsule. The thin fibrous capsule around the collagen membranes was composed of loose connective tissue fibers with the presence of very few macrophages, giant cells and other inflammatory cells. The fibrous capsule around the PTMC membrane was also composed of loose connective tissue fibers though appeared thicker and showed more invasion of macrophages and giant cells compared to the collagen treated animals. The macrophages and giant cells appeared to erode the surface of the PTMC membrane by phagocytosis (Fig. 2b, d).

At 2 weeks high numbers of blood vessels were present at the tissues surrounding the PTMC membranes and especially located in the tissues filling the defects covered with the PTMC membranes. By contrast the animals treated with the collagen membranes showed blood vessel formation mainly localized *in and throughout* the membranes, as well as in the surrounding tissues.

Osteoid (bone) formation was already observed after 2 weeks in both the collagen and PTMC membrane treated groups. Interestingly at 2 and 4 weeks, two different patterns of bone formation were identified. The animals treated with the collagen membrane tended to form osteoid and new bone throughout the defect by forming bony islets (Figs. 2, 3). By contrast the PTMC membrane treated animals showed osteoid and new bone formation originating from the defect borders. The pattern of new bone formation was found similar for both groups after 12 weeks, since the majority of the sections by then showed complete bridging of the defect with newly formed mature bone.

### Histological analysis

**Fig. 4:**
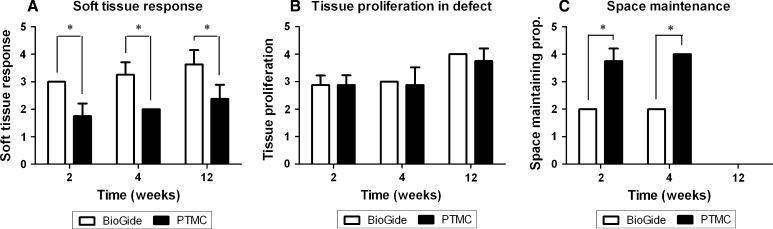
**a** Histological scoring results for the soft tissue response to the collagen and PTMC membrane after 2, 4 and 12 weeks. *Error bars* represent means ± standard deviation for *n* = 4, *p < 0.05. **b** Scoring results for the proliferated tissue at the mandibular defect site. *Error bars* represent means ± standard deviation for *n* = 4, *p < 0.05. **c** Scoring results for the space-maintaining properties for both membranes. At time = 12 weeks the space-maintaining properties could not be assessed because of closure of the mandibular defects and degradation of both the membranes. *Error bars* represent means ± standard deviation for *n* = 4, *p < 0.05

Figure 4a shows the data of the histological rating of the soft tissue response to the collagen and PTMC membrane, respectively. Statistical analysis of these data revealed that after 2, 4 and 12 weeks significant differences existed between the tissue response towards the used membranes (*p* < 0.001, *p* < 0.001 and *p* = 0.006, respectively). The tissue reaction to the PTMC membrane shows a higher density of inflammatory cells after 2, 4 and 12 weeks compared to the collagen membrane. For both membranes the score increased at 12 weeks, which means that there was a reduction in inflammatory cell density, and the number of giant cells, and an increase in vascularization of the surrounding tissue.

The data on tissue formation at the defect site, shown in Fig. 4b, was similar for both membranes. After 2 and 4 weeks the majority of the sections showed amounts of osteoid and bone formation in the defects for both membrane treated groups. Between 4 and 12 weeks the newly formed bone had matured and after 12 weeks extensive amounts of mature bone were found for both membrane groups. There were no statistical differences in the tissue proliferation at the defect sites between the collagen and PTMC treated animals (*p* = 0.467). Moreover, after 12 weeks mature bone with bone marrow had formed at the defect sites, whereas after 4 weeks bone could be assessed as immature.

Figure 4c presents the data on the space maintaining properties of the two membranes. The PTMC membrane showed a distinctively maintained space at the level of the mandibular defects, between the buccal and lingual placed membranes after 2 and 4 weeks (Figs. 1, 2). This space was filled with either connective tissue, osteoid or newly formed bone depending on the time of evaluation, respectively 2, 4 or 12 weeks. The collagen membranes by contrast showed no space maintaining at all after 2 and 4 weeks. After 12 weeks the space maintaining properties of both membranes could not be assessed for two reasons: firstly, because bone had bridged the majority of the defects, and as a result there was no ‘space’ left to be assessed, and secondly because of the extensive degradation of both membranes. The collagen membranes could not be discerned anymore due to degradation and similarity to the host connective tissue and for the PTMC membranes only some phagocytosed particles were observed. As a consequence the space maintaining properties could not be assessed, and score ‘0’ was assigned to all specimens of the 12 weeks group.

## Discussion

In the present study a rat model was used to investigate the soft and hard tissue response to a barrier membrane composed of solid PTMC. Furthermore the space maintaining properties were assessed, as space maintaining is an important factor in GBR. While copolymers prepared from lactide, glycolide and TMC have been intensively investigated and are now in clinical use [17–19], this is the first time barrier membranes prepared from TMC only have been applied in GBR.

Critical size rat mandibular defects were selected as the orthotopic model to evaluate the PTMC membranes. Previously performed studies have shown that 5.0 mm circular rat mandibular defects are of critical size and therefore will not heal spontaneously during the lifetime of the animal [25, 26].

The benign soft tissue response to PTMC membranes indicated that PTMC membranes are promising materials for use in GBR techniques. The thin cellular capsule around the PTMC membranes implies that the host has initiated the foreign body reaction that is observed with many implant materials, and at the same time still is involved in a response at the interface with the implanted material. The results after 2, 4, and 12 weeks show that the soft tissue response to the PTMC membrane is accompanied by a larger influx of macrophages, giant cells and other inflammatory cells concentrated at the tissue–implant interface when compared to the collagen membrane. However, this reaction can be regarded as a normal tissue response to the PTMC, as the degradation is characterized by a surface erosion process [22, 27]. The surface erosion process attracted cells involved in the foreign-body reaction and degradation to the tissue–implant interface. This is in accordance with the results of previous studies, where also an influx of macrophages and formation of foreign-body giant cells was observed at the tissue–implant interface [22, 27]. The foreign-body giant cells were only transiently present and disappeared with increasing implantation time. Therefore, it can be concluded that the tissue reaction (i.e*.* foreign-body reaction) induced by the PTMC membrane was mild and transient.

The tissue response to the collagen membrane was characterized by an even milder reaction, possibly due to its resemblance to native collagen. The degradation profile is characterized by a macrophage and polymorphonuclear leukocyte associated degradation as well as an enzymatic degradation profile [28, 29].

The histological evaluation of the proliferated tissue at the defect site showed for both membranes that increasing amounts of bone had formed with time, witnessing the defect closure with increasing implantation time. Recent quantitative analysis of the amount of bone formed in rat mandibular defects covered using PTMC and collagen (and e-PTFE) membranes had shown that significant amounts of bone were formed, without statistically significant differences between the membranes [16]. This implies that use of the PTMC membrane facilitates in bone regeneration in critical size defects in rats. These results suggest that the PTMC membrane is biocompatible with bone and assists in promoting bone regeneration in critical size defects.

As mentioned before, two different patterns of bone formation were observed. The bone formed beneath the PTMC membranes tended to grow from the defect borders towards the centre of the defect. This way of defect closure has been described often in literature and seems to be the most common in the formation of new bone when non-osteoinductive biomaterials are applied [30–32]. The collagen membrane showed bone formation within the membrane and throughout the defect originating from bony islets and the defect border. This has been previously described by Hoogeveen et al*.* in a comparative study between three different barrier membranes used in GBR [33]. A possible explanation for the formation of these bony islets could be that the animal derived natural collagen has osteoinductive properties. Nonetheless, the different bone formation patterns did not show statistical differences regarding the tissue proliferation at the defect site between the two membranes for the different time periods. After just 2 weeks osteoid formation was present for both membranes, again indicating that the PTMC is a bone biocompatible material that allows for bone formation in the space in between the membranes.

Space maintaining properties of the barrier membrane are important in GBR to lead to predictable bone formation, by providing space for the blood clot and new bone formation in the defect. During the implantation procedure it became clear that the handling and space maintaining properties of 0.3 mm thick PTMC membranes were superior to the collagen membrane. This was confirmed by the results of the histological evaluation after 2 and 4 weeks. Compared to the collagen membrane the PTMC membrane exerted better space maintaining properties.

The space maintaining properties of the membranes after 12 weeks could not be assessed for two reasons. Firstly, since both the PTMC and collagen membrane had almost completely resorbed, and therefore had lost their mechanical strength resulting in a complete loss of space maintaining properties. A second reason is that new bone had bridged the majority of the defects and space maintaining properties cannot be assessed without a distinct defect in the rat mandible.

An important problem with collagen membranes is that almost immediately upon implantation they loose their mechanical strength and, therefore, their space maintaining properties. The collagen membrane is therefore best used in combination with (autologous) grafting materials to achieve comparable results to non-resorbable e-PTFE barrier membranes [20, 34–36]. The better mechanical properties of the resorbable PTMC membrane should enable comparable results to those of non-resorbable e-PTFE membrane without the use of grafting materials. Before conducting clinical trials in humans, this should preferably be evaluated in a next study where deeper defects with larger volumes are created. This will emphasize the importance of the space maintaining properties of the membranes. A model such as the one described by Oh et al*.* [32] would be useful as they created buccal dehiscence defects in the jaw bone around dental implants measuring 4 mm in height from the crestal bone, 3 mm in depth from the surface and 3 mm in width in dogs. In this model the membranes space maintaining properties can be better assessed as larger volumes of deficient bone are involved, also its biological behaviour around dental implants and more importantly its behaviour towards exposure to the oral environment can be assessed. Nevertheless, the present study demonstrated histologically that the PTMC membrane allows bone growth and therefore makes it suitable for use in GBR techniques.

Finally, the general opinion in medicine is that (resorbable) animal derived materials should be replaced by similarly performing (resorbable) synthetic materials when available, in order to avoid possible transmission of disease [3–5]. In this respect, the application of PTMC as a barrier membrane in GBR techniques could be a step forward as an alternative for the animal derived collagen membranes.
